# Obituary

**Published:** 1878-12

**Authors:** 


### Obituary.

Dr. Lucius Clark, of Rockford, died at his residence Tuesday evening, Nov. 5, 1878, after an illnes of nearly three months, in the 66th year of his age. On August 22d he suffered a paralytic stroke, and on Monday evening, Nov. 4, he began failing rapidly and continued to decline until Tuesday morning, when he became unconscious, and remained in that condition until he expired.

Dr. Clark was of Massachusetts parentage ; was born in Amherst, June 10, 1813, and was educated there. He pursued his medical studies at Berkshire Medical College, Mass., and at Geneva Medical College, N. Y., where he received the first diploma given by that institution. He practiced in Western New York, at Marion, Palmyra and Chili, for 10 years ; finally removed to Rockford in 1845, where he resided and was in active practice until his death. He was a member of the American Medical Association, and of the Illinois State Medical Society, and during the war was in the field a short time as president of the board of examining surgeons for the State of Illinois. He was a trustee of Rockford Female Seminary from its organization. In 1836 he married Julia A. Adams, of Hinsdale, Mass., who died in 1861. In 1864 he married Charlotte M. Townsend, of this city.

The deceased had four brothers, two of whom are physicians, Dr. E. N. Clark, of Beloit, and Dr. Asahel Clark, of Detroit, Michigan. He has two sons who are also practicing physicians, Dr. L. A. Clark, of San Francisco, Cal., and Dr. D. S. Clark, of Rockford. He also leaves a wife and two young daughters. Dr. Clark having been a resident of Rockford for 33 years, was widely known, and was very highly esteemed by all with whom he came in contact. A wide circle of friends mourn his loss.

At a special meeting of the Rockford Medical Association, Nov. 6, 1878, the following resolutions were unanimously adopted:

WHEREAS, This Association has learned with deep regret of the loss of one of its oldest and most valued and respected members, in the death of Dr. Lucius Clark, who for a third of a century has been a zealous co-worker in our midst;

*Resolved*, That we recognize in this event the departure from among us of one who for many years was an ornament to this Society, to the profession of medicine, and to the community at large.

*Resolved*, That we extend to his afflicted family in this their bereavement the tribute of our earnest sympathy, and assure them that we will ever cherish his memory in kindest regard.

*Resolved*, That as a token of our respect for the departed, this Association, as such, attend the funeral.

*Resolved*, That a copy of these resolutions be presented to the family, and sent to the local press, and CHICAGO MEDICAL JOURNAL AND EXAMINER, for publication.

A. M. CATLIN, *President*,
H. W. TEBBETTS, *Secretary*.

AT a meeting of the senior class of the Chicago Medical College, the following resolutions were passed:

WHEREAS, In view of the loss we have sustained by the decease of our friend and classmate, Oliver L. Latta, and of the still heavier loss sustained by those who were nearest and dearest to him; therefore, be it

*Resolved*, That it is but a just tribute to the memory of the departed to say that in regretting his removal from our midst we mourn for one who was in every way worthy of our respect and regard.

*Resolved*, That we sincerely condole with the family of the deceased on the dispensation with which it has pleased Divine Providence to afflict them, and commend them for consolation to Him who orders all things for the best, and whose chastisements are meant in mercy.

*Resolved*, That copies of this heartfelt testimonial of our sympathy and sorrow be forwarded to the parents of our departed friend, to the Goshen papers, and to the CHICAGO MEDICAL JOURNAL AND EXAMINER, for publication.

C. H. BRYANT,
C. H. FEYERS,
J. M. WILCOX.
NOV. 6, 1878. *Committee*.

